# A knowledge implementation model in health system management based on the PARIHS model

**DOI:** 10.1186/s12961-022-00874-7

**Published:** 2022-06-16

**Authors:** Ghanbar Roohi, Mohammad Ali Jahani, Zeynab Farhadi, Ghahraman Mahmoudi

**Affiliations:** 1 Healthcare Services Management, School of Nursing and Midwifery, Golestan University of Medical Sciences, Gorgan, Iran; 2 Social Determinants of Health Research Center, Health Research Institute, Babol University of Medical Sciences, Babol, Iran; 3 Hospital Administration Research Center, Sari Branch, Islamic Azad University, Sari, Iran

**Keywords:** Knowledge management, Implementation science, Evidence-based healthcare management

## Abstract

**Background:**

The gap between knowledge and practice, along with postponing or not implementing research findings in practice and policy-making, is one of the reasons for low-quality services. Hence, this study aimed at presenting a model of knowledge implementation in health system management in Iran.

**Methods:**

The present two-phase study was first performed qualitatively using a directive content analysis approach based on the Promoting Action on Research Implementation in Health Services (PARIHS) model. The researchers extracted the barriers and facilitators by conducting semi-structured individual interviews. Then, in a three-stage Delphi study, 25 health experts determined the barrier removal strategies. Data were analysed using MAXQDA10 software.

**Results:**

The content analysis of the interviews led to the emergence of 1212 codes under three categories of evidence, context and facilitation. The findings indicate that health managers make fewer decisions based on research findings. Instead, they make decisions regarding the experiences of service providers and organization data. In addition to the subcategories in the PARIHS model, the researchers extracted political, social and administrative factors under the context category. The relationships between the features of evidence, context, facilitation, barriers and strategies were presented in the final model.

**Conclusion:**

The presented model comprehensively emphasizes the evidence resources, context preparation, and facilitation of the knowledge implementation process.

## Background

Quality of care delivery is one of the major principles of health policy, currently on the agenda of policy-makers at the national, European and international levels [1–4]. The rapid progress of medical sciences and new technologies, on the one hand, and the increase in people’s level of knowledge and improvement of their economic status in recent years, on the other hand, has led to an increase in people's expectations of modern healthcare services. Millions of dollars are annually spent on healthcare services, whereas they do not meet the required quality [5, 6]. The gap between knowledge and practice, along with postponing or not implementing the research findings in practice and policy-making, is one of the main reasons for the poor quality of health services worldwide [6, 7]. As a result, the health system will have poor health outcomes, inequality and waste of time and financial resources as long as it does not apply research evidence in practice [8]. In addition to producing knowledge, universities should facilitate and encourage appropriate approaches to evidence-based practice by conducting research [9]. A better understanding of decision-making processes and common resources helps the decision-makers minimize preventable mistakes and increase the shared decision-making [10].

One of the most significant clinical research findings and healthcare services is the failure to translate research into practice and policy. To understand the inadequacies of knowledge implementation, international policies and research foci have been directed toward how to reduce the gap between science–practice–policy-making [6]. In this regard, Canada's Health Research Foundation, the British Medical Association, and the National Institutes of Health have made great efforts that have led to models of knowledge implementation [11, 12].

Knowledge implementation is a process requiring interactive engagement and interaction between researchers and research users and strongly relies on the characteristics of the end-users [13]. Therefore, the required data for designing and developing applicable models must be obtained in the context of knowledge production [14]. Due to the complex process of knowledge implementation, the use of multilevel approaches is necessary to present effective factors in the form of conceptual frameworks and models [15]. In this regard, various presented models mainly concentrate on applying research results in healthcare services; they ignore preparing the context for knowledge implementation [16, 17]. The Promoting Action on Research Implementation in Health Services (PARIHS) model, developed by Kitson et al. in 1998 and revised over years of study, is one of the conceptual frameworks. This model is the outcome of the interaction of the three fundamental elements of evidence, context and facilitation. Evidence refers to research results, experiences of service providers and recipients, knowledge and information available in the organization. Context consists of culture, leadership and evaluation, and facilitation comprises the goal, role and skills of facilitating individuals or organizations [18]. A study by Ward et al. at the Iowa Department of Public Health was conducted with the aim of improving the quality of team strategies and tools to enhance performance and patient safety (TeamSTEPPS) with a PARIHS framework in 13 small rural hospitals. The findings of the study identified two of the three elements of the PARIHS model (context and facilitation) as important factors in successful implementation [17].

Consequently, an efficient and expressive model guarantees a better selection of the needed interventions and ensures their increased effectiveness [19]. One of the most widely used methods for constructing and developing conceptual frameworks or models is qualitative content analysis, using real-world data [20]. Therefore, the researchers conducted this study to present a model for knowledge implementation in the Iranian health system management.

## Methods

### Study design

The present study is an applied study conducted in two phases.

### First phase

First, we used a qualitative approach using directive content analysis; this deductive content analysis included assessing the previous theories or developing the existing ones [21]. Coding was based on previous findings; however, the researchers applied a strategy of immersion in the data during the data analysis and allowed the categories to emerge from the heart of the data [22]. In this study, the dominant method for sampling has been a goal-based approach with maximum diversity in the disciplines aware of this. We have included individuals who can provide the most comprehensive and relevant data related to research questions in the field of existing conditions, barriers and facilitators and strategies for implementing the process of applying the knowledge of health services management in the field of health and provide the individuals with answers to the main research question. According to the research process, the snowball method was used during the research. To accomplish the study, 15 health managers and executives with at least 5 years of experience in health system management, selected purposefully with the highest diversity, participated in the interviews after obtaining consent from the ethics committee. The participants were working in medical universities and health insurance organizations. Moreover, they had at least 6 years of experience in healthcare management and were willing to participate in the interview. After scheduling an interview with the participants and obtaining their informed consent, the participants were interviewed using semi-structured open-ended questions in a quiet environment (it lasted 6 months). The interviews were conducted to search for and discover the predetermined categories and subcategories based on the PARIHS framework. The interviews began with open-ended and general questions, including “What resources of decision-making do you use to manage the system you are in charge?” and “Talk about your experiences in preparing the organization setting, the barriers and facilitators for applying management knowledge.” Then, we posed an in-depth follow-up exploratory question to illuminate the answers. The interviews lasted 60–90 minutes and continued until data saturation.

The PARIHS framework is a common multidimensional conceptual framework in the implementation of science [23]. It is the closest model of applying knowledge in the healthcare system, consisting of three elements: evidence (i.e. research results, experiences of service recipients, experiences of service providers, and data in the health system), context (i.e. culture, leadership and evaluation) and facilitation. The interaction of these elements paves the way for using evidence-based practice as the basis of this study [23]. To ensure the accuracy and reliability of the data, the researchers used credibility, transferability, confirmability and dependability as the criteria for scientific accuracy in qualitative research presented by Lincoln and Guba [24]. Therefore, using the PARIHS theoretical framework, the maturity of implementation science increases [23].

We interacted with the participants and spent substantial time collecting and analysing the data. This helped us to ensure the credibility of the study. Moreover, we obtained the participants' verification during the interview and after analysis in a focus group meeting to discuss our conclusion and interpretation. After receiving feedback from the supervisor and advisor, the researchers reviewed the data, findings and conclusions. Finally, the researchers obtained comments from colleagues and experts on knowledge implementation and continuously compared the interpretations with the raw data in the report of the findings of the content analysis.

To achieve transferability, we clearly and meticulously described all the steps taken in designing, implementing, analysing and interpreting the findings. Regarding the dependability of the findings, in addition to reporting accurate and correct quotes from the interviewees, the opinions of several peers not involved in the research were taken into account to match the data and findings. Besides, during the joint meetings, the research group reviewed the extracted codes in content analysis. Finally, accurate recording of all research processes, note-taking during and after the interviews, and reminders guaranteed the confirmability of the findings.

The dependability of the data was confirmed by reading and recoding the transcripts of the interviews several times to reach common codes. After coding, we gave several initial interviews to qualitative researchers and included their suggestions in revising the codes. To increase the dependability of the data, two 2-h group discussion sessions were held with 11 participants who had taken part in previous interviews. This helped us confirm the results of interviews and determine the barriers and facilitators after extracting the codes and the main and secondary elements.

To analyse the content in the coding process, the researchers, after listening to the interviews several times and translating them, focused on the texts and entered the codes into MAXQDA10 software based on the PARIHS framework. It was then reviewed several times by the research team and, if necessary, referred to the participants again. After these stages, subclasses, classes and the main theme were obtained; the research team participated in all these stages.

The research team was made up of researchers specializing in qualitative and evidence-based research.

### Second phase

The three-stage Delphi method was employed with 25 faculty members and managers of healthcare services with at least 5 years of experience in the Iranian healthcare system. First, the barriers extracted from the content analysis and group discussion were sent to the panel members through email with prior arrangements. They were asked to present strategies to overcome these barriers. After receiving the results, we summed them up and omitted the duplicates.

Afterward, we arranged the extracted strategies based on their effectiveness, implementability and accordance with the conditions of the health system. We sent them to the participants to obtain their agreement or disagreement with the strategy. After reviewing the results, we extracted the strategies. In these two stages, the criterion for accepting the strategy was an agreement of more than 70% in the participants’ opinion.

In the third stage, we sent the extracted strategies from the previous stage to the panel to prioritize them based on the second-stage criteria. After we received the results, we found that the number of participants at the beginning of the study was 33, whereas only 25 replied to the emails [25, 26].

Finally, the research team analysed and prioritized the extracted strategies. To do so, they added the priority order number given by the participants and divided them by the number of priorities. The obtained scores were used to determine the first, the second and the third priority strategies. Then, considering the required evidence, context conditions, barriers, facilitators and implementable strategies for knowledge implementation, we identified its characteristics and presented a conceptual model of knowledge implementation in health system management.

## Findings

In this study, 51 people participated, including health system managers, faculty members, and heads of the Department of Health Services Management (Table 1).

**Table 1:** Demographic characteristics of participants in knowledge implementation in health system management

Type of participation	Mean age by year	Gender		Mean work experience	Education degree			Total
		Male	Female		Masters	PhD	Medical specialist	No
					No	No	No	
Individual interview	54.2	13	2	27.4	2	6	9	17
Group discussion	53.1	9	2	26.9	–	5	6	11
Delphi method	46.6	20	5	21.9	–	21	4	25
Total	–	42	9		2	32	19	53

After analysing the interviews, removing duplicated codes and merging similar cases, we obtained 1222 codes, 11 subcategories and three main categories. All the obtained categories conformed to the PARIHS framework, although we added two subcategories of political, social and administrative factors and appointment of managers. The results indicated that these factors were effective in knowledge implementation in health system management (Table 2). Some of the results of this study have already been published in an article examining the status of knowledge implementation in health system management [25].

**Table 2:** Extracted codes from the interview of knowledge implementation in health system management

Main categories	Subcategories	Number of assigned codes (comments)
Evidence for managerial decision making	Research	11
	Experiences of service providers	13
	Opinions of service recipients	35
	Internal data and information of the system	65
	Total	124
Context for implementing managerial decisions	Culture	264
	Leadership	173
	Evaluation	98
	Political, social and administrative factors	66
	Appointment of managers	108
	Total	864
Facilitation for implementing the managerial decisions	Skills and characteristics	72
	Purpose and role	38
	Total	100
Sum of all extracted codes		1222

The results of the barriers extracted from the transcripts of interviews and focus group discussion indicate there are 38 barriers to the knowledge implementation process in health system management. The participants proposed 430 strategies in the first stage of the three-stage Delphi method; however, the number of strategies was reduced to 271 in the second stage. Finally, in the third stage, 135 strategies were approved by the participants as final strategies for removing the existing barriers in the route of knowledge implementation in the health system management of the country. The section on barriers and strategies for knowledge implementation was already published in an article entitled “Barriers and Strategies for Implementing Knowledge into Health System Management: A Qualitative Study” [26].

**Fig. 1:**
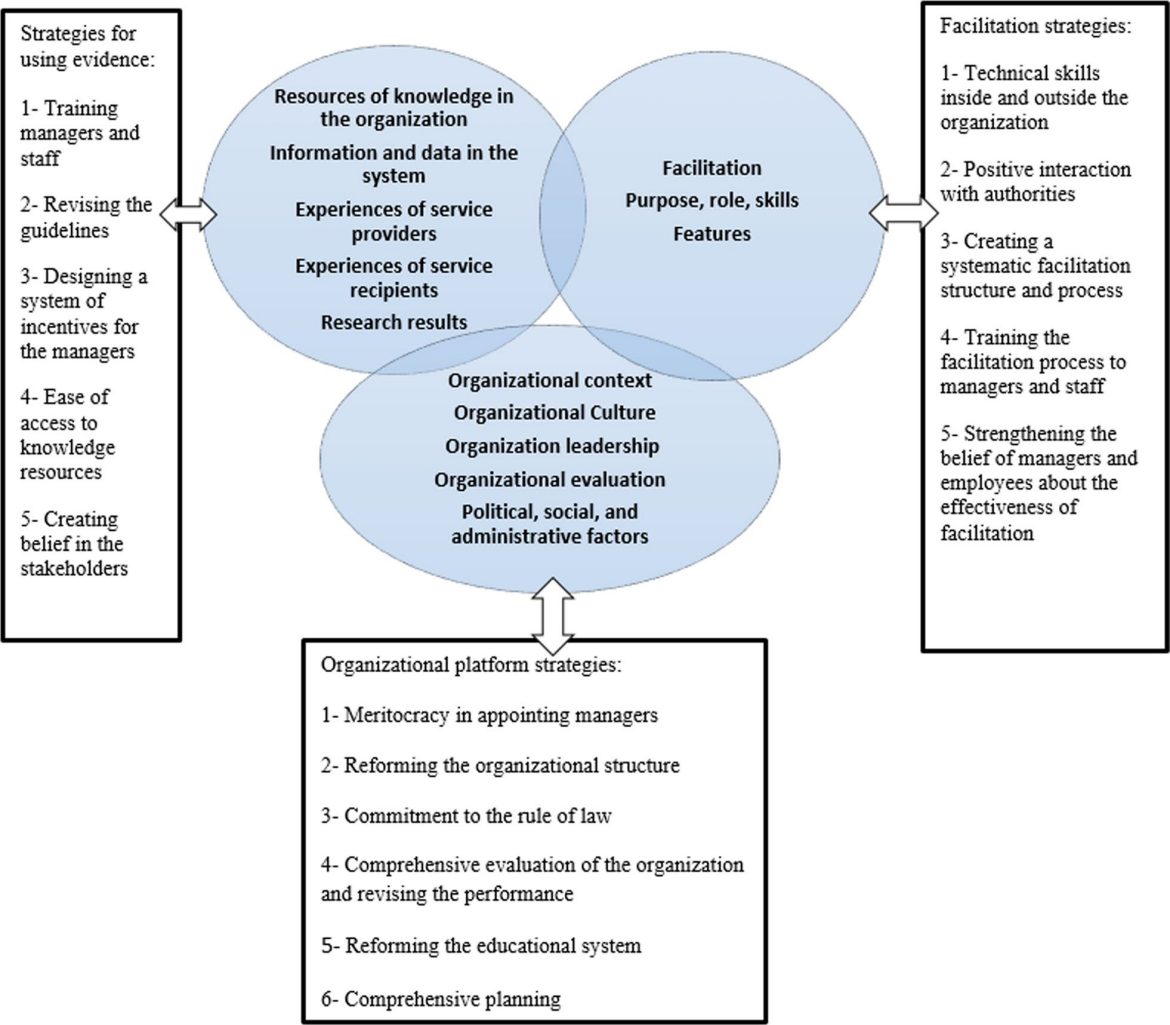
Knowledge-based health system management model

After reviewing and merging the results of content analysis and the three-stage Delphi method, the research team extracted the final model, including the three elements of evidence, context and facilitation. The evidence consists of four sources (research results, experiences of service providers and recipients, and data and information available in the organization). There were also several barriers in the knowledge implementation path obtained from the four sources. After the research team reviewed the strategies, they extracted the final model containing the final strategies for producing evidence, creating a suitable context, and facilitation in the process of knowledge implementation in health system management (Fig. 1).

### Designing a system of incentives for the managers

The results of this study revealed barriers such as the insufficient engagement of managers in management research and the lack of applying a combination of four sources of knowledge in decision-making. Therefore, designing a system of incentives for the managers’ strategy was suggested in the four sources of knowledge and inserted in the model. One participant noted:

*Forcing managers to use this evidence in the workplace, for example taking into account using such experiences in their annual promotions, can encourage them to make evidence-based decisions.*

### Ease of access to knowledge resources

Managers' workload and lack of time to devote to search for evidence are two barriers in the route of knowledge implementation. Therefore, ease of access to a knowledge resources strategy was included in the model by creating a system of recording experiences and preparing a summary of research results. Another participant observed:

*The summary of the research results should be made available to managers, or a special database should be created for them. Besides, some sessions should be held to review the research results.*

### Training the managers and staff

The training of the managers and staff strategy is included in the model due to the managers' insufficient mastery of information technology (IT), searching for sources of knowledge, and training in management skills that were among the barriers:

*Training is required; training courses should be held for managers and employees to fully master IT, searching the databases, management and communication skills, etc.*

### Engaging the stakeholder and creating belief among them

Moreover, the results revealed that if the employees are not motivated enough, they resist change. Additionally, the managers’ belief in the effectiveness of applying the research results is weak. Therefore, we proposed the strategy of engaging the stakeholder and creating their belief in the model. One of the participants noted:

*It should be part of the managers' belief that using the previous managers’ experience is one of the sources of evidence.*

### Revising the guidelines

Revising the guidelines, as the existing data and information, is included as a strategy in the model because they interrupt and oppose each other:

*A committee must be established to revise the guidelines to avoid falling into the trap of the circulars. We should benefit from international scientific standards and make the guidelines in line with the scientific basis.*

Moreover, the findings of the qualitative part of the study showed that in addition to culture, leadership and evaluation, which are consistent with the PARIHS framework, political, social and administrative factors, arising from the specific conditions of different societies, were also present in the context of the model. The results indicated that there were some barriers in this section, and the participants in the Delphi stages suggested the strategies outlined below for overcoming them.

### Reforming the structures and processes of the educational system

To eliminate the problems related to conducting experience-based management and lack of conformity between the content of education and real-world needs, we presented reforming the educational structure strategy in the proposed model:

*Reforming the content of education is one of the issues that those in charge of medical science education in the country should constantly address and train human resources according to the needs of the country.*

### Reforming the structure of the health system

The results of our study showed that the inflexible, complicated and centralized structure of the health system, along with an island view, has hindered the application of knowledge in the organization. Therefore, we embedded the strategy of reforming the organizational structure in the model:

*The existing organizational structure in the country's health system has been developed since three decades ago. The organization needs an up-to-date structure.*

### Comprehensive planning in the health system

According to the results of this study, the activities of the health system do not conform to its objectives. That is, scarce resources are sometimes allocated based on personal preferences. Therefore, comprehensive planning was proposed as a strategy in this part of the model:

*Over the past years, the health system has acted mainly as an island…. Therefore, interdepartmental cooperation to promote the health of the society is one of the decision-making priorities of managers and policy-makers in the health system.*

### Meritocracy in appointing managers

The results of this study demonstrated that in some cases, appointing the managers is not based on the regulations, and they do not have the necessary qualifications due to instability:

*The conditions of appointing and approving the scientific and executive qualifications of a health manager must be revised.*

### Commitment to the rule of law

Our study showed that the political recommendations, provincial and national authorities, and replacement of regulations with relations make barriers in the executive processes of the organization. Therefore, commitment to the rule of law was proposed as a strategy to overcome these barriers.

The third element of this model is facilitation in knowledge implementation. It results in interaction between the context and the four sources of knowledge to prepare the conditions for knowledge implementation. As the results of this study presented, the health system managers are not that aware of this issue and are mostly appointed through lobbying and making a relationship with the authorities. The participants in the three-stage Delphi method suggested the following strategies to eliminate the barriers.

### Creating a systematic facilitation structure and process

According to the results, the lack of organizational structure in the facilitation category was the main problem in this element. The results of the Delphi method added the strategy of creating a systematic facilitation structure and process in the model:

*It is one of the most important requirements in setting the processes in the health system, so creating an appropriate structure will be possible when health system planners and policy-makers use evidence in their jobs.*

### Training and promoting the managers’ and staff’s belief about the effectiveness of facilitation

Since the results of this study indicate that managers have little knowledge and belief about facilitation, we added the strategy of training and promoting belief in the stakeholders to the model.

### Positive interaction with the authorities

As the results showed, one can transform a weakness into an opportunity by positively interacting with the authorities. Considering the social characteristics in Iran, interaction with authorities is a strategy presented in the model.

### Model features

The model obtained from this study has three separate sections of evidence, context and facilitation, and barrier removal strategies. In the evidence element, health system managers must create the conditions for knowledge production from the four sources of knowledge and integrate them into the decision-making if they are valid.

In addition to the subcategories of culture, leadership and evaluation in the context, political, social and administrative factors are embedded in the model. Hence, health system managers must provide the necessary basis for using the knowledge of management.

The facilitation section acts as an umbrella that covers all the processes of producing and implementing knowledge. It also provides the context to help the managers to apply knowledge.

The results of this study revealed that there are several barriers to knowledge implementation in health system management. This model provides practical strategies to eliminate these barriers. Consequently, we can claim that the present model, with a comprehensive view to various sources of knowledge as evidence, context and facilitation and providing practical strategies to remove barriers, can contribute considerably to establishing an evidence-based health system management.

## Discussion

The results showed that the final model includes three parts: evidence-utilizing strategies, organizational context, and facilitation strategies. Resources of knowledge in the organization consist of research results, experiences of service providers, experiences of service recipients, and information and data in the system. Health system managers can apply them using appropriate strategies. Since applying knowledge in the health management domain is a complex and interactive process, assessing the value of knowledge management is one of the main concerns of today's organizations. As with other intangible assets, the reliability of measuring knowledge management in organizations is a matter of debate. Because internal evaluations by managers may be biased, on the other hand, evaluations performed by third parties may not be accurate due to lack of access to internal knowledge assets. Therefore, knowledge is valued as an intangible asset and has no good market value. In this regard, there is no market structure that can regulate the evaluation of knowledge. It is believed that knowledge producers should pay attention to knowledge users, their needs and the organizational context [27–29]. Similarly, Metzler et al. believe that knowledge implementation in health management has a complicated structure [30]. Moreover, Jacobson et al. see knowledge implementation as a set of strategies and communication or interaction between stakeholders [31]. Since the present model has a social approach and emphasizes the interaction between the stakeholders, it can guide the knowledge produced from different sources to provide quality services using proposed strategies. The ultimate message of this model is the interaction between its components.

According to the proposed model, this study has three parts: evidence-utilizing strategies, organizational context, and facilitation strategies. The model presented by Visram et al. includes definitions and concepts, subject process, and people [32]. On the other hand, the framework posed by Ellen et al. has extracted environmental components, producing new knowledge, engendering belief in the users in applying the research findings and results evaluation [16]. These two models are similar to ours in terms of using the stakeholders’ opinions and considering the context; however, they differ in using different sources of knowledge. The Canadian National Institute model emphasizes determining research priorities, synthesizing knowledge, and a knowledge dissemination process and its application [33]. On the other hand, the Phillips model focuses on three dimensions of research, that is, knowledge research, teaching and learning [34]. Other models highlight implementing research results and designing research [16, 17, 19, 34]. However, the present study emphasizes the necessity to produce valid knowledge to be used by the users. Unlike other models, this model emphasizes the managers' use of four sources of knowledge. Using the experiences of the service recipients and providers, data and research results are necessary for this model. Hence, the strategies can contribute to eliminating the barriers.

The present model has three elements: evidence-utilizing strategies, organizational context strategies, and facilitation strategies. It follows the frameworks by Stetler et al. and Kitson et al. regarding the content and application. They believe that the successful application of knowledge is the result of the interaction of the three mentioned elements [7, 15]. However, the model presented in this study included the political, social and administrative factors subcategory, along with culture, leadership and evaluation in the organizational context strategy. Considering the social conditions, the influence of factors outside the organization is natural in knowledge implementation. The difference between the present study and the PARIHS framework is the strategies presented for eliminating the barriers. Due to the comprehensive view of this model, adding the strategies has highlighted the strength and ease of implementation of this model. Furthermore, the four sources of knowledge (information and data in the system, experiences of service providers, experiences of service recipients, and research results) are encompassed in the present model, and there has been an emphasis on using them in an integrated manner. Similarly, Brouwers et al. and Rycroft believe that research knowledge per se cannot address the complicated issues in the health system [18, 35]. Moreover, other researchers emphasize using a combination of sources such as research results, implicit knowledge, patient preferences, and contextual data to meet real needs [36–38]. Policy-makers generally make decisions ignoring the results of research [39, 40]. Therefore, similar to the models presented in other studies, this model includes designing an incentive system to resolve this problem [39–42]. Therefore, it seems that giving incentives will motivate managers.

In addition to the three components of the context (organizational culture, leadership and evaluation), there are political, social and administrative factors in the organizational setting. Thus, other studies have addressed establishing permanent mechanisms and cooperation between researchers and policy-makers [43, 44]. They believe that political commitment by the government and strong influence in policy-making are required to support the use of evidence in policy-making [45, 46]. Given the professional features, local characteristics and the growing effects of political and social factors on the processes governing the health system, it seems that the political and social potentials can be used to facilitate knowledge implementation. On the other hand, to prevent the negative impact of these factors, strategies such as commitment to the rule of law and meritocracy can be used in appointing managers.

Similar to other studies that have mentioned structural and process barriers due to regulations, the results of this study showed that the presence of multiple and complicated guidelines prevents the organization from moving towards its goals [47, 48]. Therefore, revising the guidelines activates the organization and speeds up the activities in the present model. As the results of this study demonstrated, managers’ lack of access to knowledge resources and workload are among the barriers that prevent knowledge implementation. The results of other studies confirm these findings and emphasize barriers such as lack of time (having multiple responsibilities and attending meetings) to apply knowledge [49], insufficient skills and newness to work processes [50, 51], and the language of the journal that is mainly English [52]. To eliminate these barriers, it is necessary to provide managers with a summary of knowledge resources. Furthermore, the experience of stakeholders should be documented on a website to facilitate using them. Therefore, the researchers proposed establishing a learning organization, that is, training the managers and staff, to eliminate these barriers.

Similar to the study by Kernohan et al., the results of our study showed that the lack of a system for recording experiences and information is one of the barriers to knowledge implementation [53]. Since the barriers to knowledge implementation are enduring, the cooperation of academic institutions and policy-makers can resolve them [54, 55]. However, most of these barriers are related to the organizational context, including lack of resources, infrastructure and access to computers and the Internet [56]. Therefore, it is necessary to reform the structure and processes of the health system to remove these obstacles. This can provide the necessary context for recording the data and producing the required knowledge while fairly preparing the sources. Hence, reforming the educational structure to remove educational barriers has also been emphasized in the present model. That is, creating or changing the education structure [57], purposeful education [58], using various sources, involving stakeholders for evaluation [59, 60], and applying IT and cyberspace in evaluation [61] are counted as facilitators in applying knowledge management.

According to the results of this study, facilitation strategies are more in the form of establishing personal relationships with influential people. Therefore, there is no specific system to facilitate knowledge transfer in this regard, and interpersonal communication works better [53, 62]. Healthcare policy-makers have little knowledge and belief about facilitation and do not consider it feasible [63, 64]. Therefore, anticipating a systematic structure is necessary to act as an umbrella and cover the other two elements (i.e., evidence and context) and help the evidence-based management in the health system with the strategies.

### Research limitations

Similar to any other study, this research is subject to some limitations. Considering the existing situation of the health system, the implementation of this study is difficult and time-consuming because the researchers used the existing data and experts’ comments to develop the systematic structures and processes, including “designing an incentive system for managers” and “creating a systematic structure and process for facilitation” in the model. Moreover, “revising the guidelines” has been included in the model in the evidence.

## Conclusion

The model obtained from this study has elements of evidence, context and facilitation, where in the context category, in addition to cultural factors, leadership, political and social factors have also been added in the evaluation of the organization. It is obvious that the use of knowledge will be provided in the shadow of the use of facilitators and a suitable platform, in which the application of strategies embedded in the model can remove obstacles to the use of knowledge in health system management. It is recommended that health system managers and policy-makers employ these strategies and knowledge to succeed and improve services in the organization.

## Data Availability

The authors have full control over the primary data. The data analysed in this study are housed at the Hospital Administration Research Center in Islamic Azad University, Sari Branch, 7 km Sea Road, Sari, Mazandaran, Iran. According to the ethics committee approval, this dataset is subject to ethical restrictions and local data protection regulations regarding qualitative raw data, since participant privacy could be compromised. Participants did not consent to have their full transcripts made available for third parties. All relevant data for the conclusions are presented in the manuscript.
